# IL-33 and IL-10 Serum Levels Increase in MCI Patients Following Homotaurine Treatment

**DOI:** 10.3389/fimmu.2022.813951

**Published:** 2022-04-19

**Authors:** Elisa Toppi, Laura Sireno, Micaela Lembo, Nerisa Banaj, Beatrice Messina, Sedigheh Golesorkhtafti, Gianfranco Spalletta, Paola Bossù

**Affiliations:** ^1^ Experimental Neuropsicobiology Lab, Istituto di Ricovero e Cura a Carattere Scientifico (IRCCS) Fondazione Santa Lucia, Rome, Italy; ^2^ Neuropsychiatry Lab, Istituto di Ricovero e Cura a Carattere Scientifico (IRCCS) Fondazione Santa Lucia, Rome, Italy

**Keywords:** tramiprosate, inflammation, mild cognitive impairment, anti-inflammatory cytokines, interleukin-33, interleukin-10

## Abstract

Homotaurine is a potential therapeutic compound for treatment of Alzheimer’s disease (AD), but its efficacy is still under investigation. Emerging data have shown that other than neuroprotective, homotaurine is endowed with anti-inflammatory activities, though with still unclear underlying mechanisms. Inflammation plays a critical role in the pathogenesis of AD and we previously suggested that homotaurine supplementation in patients with amnestic mild cognitive impairment (MCI) plays beneficial effects associated to a decrease in the circulating levels of the pro-inflammatory cytokine IL-18. Here we report that MCI patients supplemented with homotaurine for 12 months show elevated serum levels of IL-10 and IL-33, as compared to baseline, in addition to the described IL-18 decrease. Furthermore, we observed a significant positive correlation between IL-10 and IL-33 levels after treatment but not at the baseline, underlining the effectiveness of the compound in modulating both cytokines in an inter-related fashion and in regulating the pro/anti-inflammation balance. Furthermore, the elevation of both IL-10 and IL-33 is significantly associated with an improvement of episodic memory of treated patients, as measured by the Delayed Verbal Ray Test. In conclusion, our results confirm that homotaurine treatment exerts an overall anti-inflammatory action in MCI patients, based not only on the down-regulation of pro-inflammatory IL-18, but also on up-regulation of the anti-inflammatory IL-33 and IL-10 cytokines, which in turn are associated with an amelioration of patient’s cognitive functions. Future studies should be addressed to investigate the molecular mechanisms of homotaurine anti-inflammatory activity and its therapeutic exploitation in early AD.

## Introduction

Alzheimer’s disease (AD) is a slow, progressing and incurable neurodegenerative disease resulting in loss of memory and dementia. Its distinctive neuropathological manifestation is a brain load of misfolded proteins, including aggregated species of amyloid beta (Aβ) peptides. Regardless accumulation of misfolded Aβ appears central in AD pathogenesis, a complex interplay among Aβ itself, abnormal tau protein and several other factors together with inflammation may concur in causing the disease, whose precise etiology remains elusive shaping a persistent lack of cure (1).

Homotaurine, or tramiprosate, is a natural amino acid first identified in different species of marine red algae with some anti-amyloid therapeutic potential in AD. It is a blood-brain barrier permeable compound, able to inhibit Aβ misfolding and oligomer formation and elongation, thus capable to provide neuroprotection against Aβ aggregation and deposition (2). When administered to patients with mild to moderate AD, homotaurine is safe and well tolerated, but the results obtained from the initial clinical trials did not show efficacy in the overall study population (3). Additional studies suggested that patients carrying the strongest genetic risk factor of AD, namely the ApoE ϵ4 allele, are the optimal target population for tramiprosate treatment (4). Of interest, the orally administrable prodrug of homotaurine ALZ-801, endowed with improved pharmacokinetic properties and gastrointestinal tolerability, was shown to fully block the formation of amyloid oligomers in the brain at the target clinical dose (5). Currently, ALZ-801 is in the late stages of clinical development for treatment of early AD patients with ApoE ϵ4 genotype (6).

Overall, homotaurine is not yet authorized as a new AD drug, but its use in the treatment of cognitive decline symptoms and for prevention of AD (7) is still considered promising, and it is currently under analysis as a nutraceutical. In fact, a number of research studies have evaluated the effects of homotaurine supplementation in the earliest stages of AD, reporting its ability to ameliorate the cholinergic transmission by modulating the inhibitory cortical activity in MCI patients (8). After 12 month of supplementation with homotaurine, an amelioration in hippocampal volume and episodic memory has been observed in amnestic MCI patients (9), while the Mini-Mental State Examination (MMSE) score was found improved from baseline at months 8 and 12 in amnestic, and at month 4 in non-amnestic MCI patients (10).

Homotaurine is also an analog of the inhibitory neurotransmitter γ-aminobutyric acid (GABA) that has a fundamental role in driving glial function towards anti-inflammatory features (11). On this basis, recent studies have proposed homotaurine as a therapeutic candidate in autoimmune and inflammatory diseases, including multiple sclerosis (12–14).

To assess the potential anti-inflammatory effects of homotaurine in early AD clinical conditions is of great importance to allow the understanding of the mechanism of action and the power of this compound to modulate the AD progression (15). In full accordance with its potential to regulate immune response, a previous study conducted in our own laboratory showed that homotaurine induces in amnestic MCI patients a significant decrease in interleukin (IL)-18 serum levels (16). Together with IL-1β, IL-1α, IL-1Ra, and IL-33, IL-18 belongs to the IL-1 superfamily and it is a pleiotropic cytokine previously associated with AD-related neuroinflammation (17, 18). While IL-1 and IL-18 are endowed with pro-inflammatory activities, IL-1Ra and IL-33 predominantly exert anti-inflammatory effects (19), thus to gain a more comprehensive picture of the patients’ inflammatory status, it is advisable to measure the levels of both pro-inflammatory and anti-inflammatory IL-1-related molecules. In particular, the alarmin IL-33 could ameliorate disease pathology and cognitive decline in AD animal models, possibly by promoting microglia capacity to phagocyte Aβ and increasing anti-inflammatory gene expression (20). In humans, IL-33 is measurable in the serum of only a fraction of subjects, who are few among healthy controls, but more numerous among amnestic MCI and even more in AD patients, and its levels are related to the preserved cognitive functions of individuals (21). Further findings suggest that IL-33 plays a complex anti-inflammatory role that may be altered in AD- and MCI-associated neuroinflammation, pointing to its possible use as a novel AD therapeutic approach (22). Eventually, IL-33 is capable to promote the production of IL-10, another molecule linked to AD neuroinflammation and involved in counteracting the damage driven by excessive inflammation (23, 24).

In order to assess the potential immunomodulating and disease-modifying effects of oral administration of homotaurine to MCI patients, here we measured both at baseline and after 12 month-supplementation, the serum levels of a panel of cytokines with either pro- and anti-inflammatory activities, including IL-1β, IL-1Ra, IL-18, IL-33, and IL-10. We also evaluated the relationships between the serum levels of these cytokines and memory functions of patients at baseline and following homotaurine supplementation.

## Materials and Methods

### Subjects

We included a group of fourteen subjects with amnestic MCI (hereafter indicated as MCI), belonging to a population previously described (16), whose clinical and demographic characteristics are summarized in **Table 1** and fully reported in **Supplemental Table S1**. More specifically, a convenience sample of 14 patients who underwent the first diagnostic assessment for memory problems in the Santa Lucia Foundation outpatient memory clinic in Rome was included (single-center study). Patients were naturalistically (not randomly) included in the study based on specific inclusion and exclusion criteria defined in the study protocol. Only patients without significant clinical factors that promote inflammation were selected. Particular attention was given to exclude patients suffering from those diseases that are known to be associated with altered cytokine production that may be common in elderly people, such as autoimmune and autoinflammatory diseases (e.g., Crohn’s disease, rheumatoid arthritis, systemic lupus erythematosus), infections and cancer. A trained senior research neuropsychiatrist made the diagnosis of MCI and two trained post-doc neuropsychologists made the cognitive assessment. Clinical examination included physical, neurological, and mental status examinations, neurocognitive tests, and brain magnetic resonance imaging. No patient had taken anti-dementia drugs in their lifetime or psychotropic drugs (i.e., antidepressants, benzodiazepines, and antipsychotics) in the previous 12 months. Inclusion criteria for MCI were: (1) diagnostic evidence of amnestic MCI consistent with Petersen guidelines (25) and (2) a Mini-Mental State Examination (MMSE) score ≥23. Specifically, for the diagnosis of MCI was required impaired performance on at least one memory test in association or not with impaired performance in at least one additional cognitive domain (i.e., praxis, attention, language, and executive functions) in the absence of functional impairment. Exclusion criteria were: (1) major medical illnesses and autoimmune and inflammatory diseases; (2) comorbidity of primary psychiatric or neurological disorders and any other significant mental or neurological disorder; (3) clinically important infection within the last 30 days (e.g., chronic persistent or acute infection, such as bronchitis or urinary tract infection); (4) implant of carotid or coronary stent or other major surgical interventions; (5) use of anti-inflammatory drugs within the last 60 days (e.g., corticosteroids or nonsteroidal anti-inflammatory drugs). (6) MRI evidence of focal parenchymal abnormalities or neoplasm. The included MCI patients underwent the first diagnostic assessment for memory problems in the Santa Lucia Foundation outpatient memory clinic in Rome and were treated with homotaurine tablets, 50 mg, QD (*quaque die*, once a day) for 2 weeks, and BID (*bis in die*, twice a day) for the next year, as recommended in medical routine in Italy (9). Blood was drawn both at the baseline and at 12 months follow-up of homotaurine supplementation. Patients’ compliance and therapeutic adherence were assessed periodically (i.e. every 3 months) in scheduled appointments with the neuropsychiatrist in the outpatient’s memory clinic of the Santa Lucia Foundation IRCCS and no deflection, neither other pharmacological and non-pharmacological treatments were recorded during the entire treatment period. Informed written consent was obtained from all subjects or, when necessary, from their proxies following the Declaration of Helsinki. The protocol was approved by the ‘‘Fondazione Santa Lucia Ethics committee.’’

**Table 1:** Sociodemographic and clinical characteristics of amnestic MCI patients.

Characteristics	Individuals (n=14)		
Age (years, mean ± SD)	75.85 ± 5.68		
Educational level (years, mean ± SD)	9.64 ± 3.85		
Gender male (n; %)	6 ; 42		
	**Baseline (T0)**	**After 12 month treatment (T12)**	**p value (T12 vs. T0)**
MMSE (score, mean ± SD)	27.071 ± 1.94	26.14 ± 1.56	*p=0.06 (n.s.)*
IADL (score, mean ± SD)	9.14 ± 3.92	9.92 ± 3.26	*p=0.25 (n.s.)*
I-RWLLT (score, mean ± SD)	26.92 ± 6.46	24.28 ± 5.9	*p=0.12 (n.s.)*
D-RWLLT (score, mean ± SD)	3.5 ± 1.7	2.57 ± 2.3	*p=0.1 (n.s.)*

MMSE, Mini-Mental State Examination; IADL, Instrumental Activities of Daily Living; I-RWLLT, Immediate recall Rey 15-Word List Learning Test; D-RWLLT, Delayed recall Rey

15-Word List Learning Test; n.s., statistically not significant.

### Neuropsychological and Functional Assessment

The Mental Deterioration Battery (MDB) (26) was used to measure performance in specific cognitive domains at baseline and the 1-year follow-up. In particular, the MDB was administered to make a comprehensive cognitive examination at the diagnostic level. To measure episodic memory performance, Rey’s 15-Word List Learning Test (RWLLT) was administered. In this task, participants are given a list of 15 unrelated words that are repeated in five different trials and asked to recall them in any order immediately afterward (immediate recall, I- RWLLT; score range: 0–75). After a 15-min interval, during which non-verbal tasks are given, the patient is asked to recall, without list repetition, as many words as possible in any order (delayed recall, D- RWLLT; score range: 0–15). The patterns of performance relative to the position of the items in the word list has been further assessed as described before (16). Briefly, recall accuracy varies as a function of the item’s position in the study list, and it is greater for words at the beginning (primacy effect) and the end (recency effect) of the list compared to the mid-list (intermediate) items (27).

Functional impairment was evaluated by assessing instrumental activities of daily living (IADL, 28). The IADL includes abilities that allow a person to live independently (e.g., food preparation, housekeeping and laundry, managing financial matters, shopping, and using a telephone). When the ability is fully or at least partially preserved a score of 1 is assigned; when the ability has been lost a score of 0 is assigned. Thus, a total score ranging from 0 (total dependence) to 8 (5 for men) for IADL (total independence) is obtained.

### Cytokine Measurement

Blood was drawn in the morning after overnight fasting, both at the baseline (T0) and at 12 months follow-up of homotaurine supplementation (T12). Serum samples were obtained by centrifugation of clotted blood and aliquots were stored at -80°C until cytokine assays.

The serum cytokine levels of IL-1Ra, IL-1β, IL-10 and IL-33 were measured using chemiluminescence-based assays from Meso Scale Discovery (MSD, Gaithersburg, MD, USA). The detection range of each molecule, as reported in the manufacturers’ claims, are: IL-1Ra, 1.7-5000 pg/ml; IL-1β, 0.15-3.8 pg/ml; IL-10, 0.14-3.7 pg/mL; IL-33, 0.59-10.3 pg/ml. All assays were performed in duplicate. Analyses were done using a QuickPlex SQ 120 instrument (MSD) and DISCOVERY WORKBENCH^®^ 4.0 software. All samples were run at the same time on a blind basis.

The levels of bioactive IL-18, corresponding to the amount of the cytokine not bound to its natural inhibitor IL-18BP, were determined as previously reported (16). Briefly, specific ELISA tests were used to measure total IL-18 (MBL, Nagoya, Japan) and IL-18BP (R&D Systems, Minneapolis, MN, USA). Detection limits for both IL-18 and IL-18BP assays was 12.5 pg/mL. The concentration of bioactive IL-18, namely the free form of IL-18 unbound to its inhibitor, resulted from calculation based on the law of mass action, considering 1:1 stoichiometry in the complex of IL-18 and IL-18BP and a dissociation constant (Kd) of 0.4 nM, as elsewhere reported in more detail (29).

### Statistical Analysis

StatView (SAS, San Francisco, CA, USA) and GraphPad (Prism Version 8, San Diego, CA, USA) software were used for statistical analyses. Because the data were referred to a small sample and did not follow normal distribution, non-parametric statistical tests were used. In particular, the differences between continuous variables at baseline and after 12 months of homotaurine supplementation were evaluated using the Wilcoxon signed-rank test, useful to compare two related samples.

In order to highlight relationships between treatment-related changes of serum cytokine levels and neuropsychological performance, differential scores (T12-T0) were calculated and delta scores were submitted, two by two, to Kendall Rank Correlation test, useful to investigate the relationship between two continuous variables with outliers. In particular, the following correlations between levels of IL-10 and IL-33 both at T0 and T12 and between the delta levels of IL-33 (T12-T0) or IL-10 (T12-T0) and the delta score of Delayed Verbal Ray Test (T12-T0) were assessed. P-values less than 0.05 were considered statistically significant.

## Results

### Modulation of Cytokine Serum Levels After Homotaurine Supplementation

The serum levels of pro-inflammatory and anti-inflammatory cytokines were evaluated at the baseline and after 12 months of homotaurine supplementation in amnestic MCI subjects and results are summarized in **Table 2** and fully reported in **Supplemental Table 2**. When pro-inflammatory molecules are taken into consideration, a significant decrease in biological active (free) IL-18 can be observed in treated patients, in line with our previously published data (16). No significant changes were monitored in IL-1β levels that, as expected, were very low and measurable in part of the individuals with high variability among them. Similarly, no significant changes were observed between baseline and follow-up levels of the anti-inflammatory IL-1β agonist IL-1Ra.

**Table 2:** Changes in cytokine serum levels of amnestic MCI patients supplemented with homotaurine.

Cytokine	Baseline (T0)	After 12 month treatment (T12)	p value (T12 vs. T0)	Overall change
*Pro-inflammatory*				
IL-1β (pg/ml, mean ± SD)	0.49 ± 0.61	0.52 ± 0.75	*p= 0.79 (n.s.)*	*-*
Total IL-18 (pg/ml, mean ± SD)	247.5 ± 143	222.14 ± 123	*p= 0.23 (n.s.)*	*-*
IL-18 BP (ng/ml, mean ± SD)	15.47 ± 5.9	16.44 ± 4.1	*p= 0.11 (n.s.)*	*-*
Free IL-18 (pg/ml, mean ± SD)	132.85 ± 71.71	117.71 ± 66.80	***p= 0.02***	Down ↓
*Anti-inflammatory*				
IL-1RA (ng/ml, mean ± SD)	439.47 ± 417.24	341.49 ± 198.60	*p= 0.27 (n.s.)*	–
IL-33 (pg/ml, mean ± SD)*	5.88 ± 5.46	11.13 ± 11.59	***p= 0.04***	Up ↑
IL-10 (pg/ml, mean ± SD)	0.41 ± 0.34	1.64 ± 2.24	***p= 0.04***	Up ↑

IL-18 BP, Interleukin-18 Binding Protein; Free IL-18, IL-18 BP-unbound IL-18; IL-1RA, IL-1 Receptor Antagonist; n.s., statistically not significant

*Measurable only in serum of 5 patients out of 14. Statistically significant p values are reported in bold.

**Figure 1:**
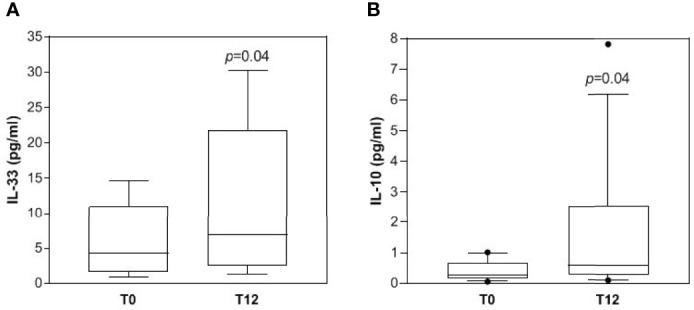
Changes of serum cytokines following homotaurine supplementation. IL-33 (**A**, n=5) and IL-10 levels (**B**, n=14) were measured as described in patients’ serum at baseline (T0) and after 12-month homotaurine treatment (T12). Distribution of data and median values are reported in each box plot.

IL-33 levels were found to be above the lower limit of detection in the assay (0.53-0.60 pg/ml) only in a fraction of MCI patients (5 out of 14), in full agreement with data reported from other authors (21). Notably, patients with measurable levels of IL-33 showed a statistically significant increase of the cytokine after homotaurine treatment, as compared to the baseline (p=0.046; **Figure 1A**).

Finally, in the whole group of patients the serum levels of IL-10 resulted significantly elevated after 12 months of homotaurine supplementation, as compared to baseline (p=0.041; **Figure 1B**). A similar elevation, though not statistically significant, was also observed in the fraction of patients with measurable IL-33 levels (IL-10 mean pg/ml: T0 = 0.58 ± 0.40, T12 = 2.50 ± 3.06; n=5).

### Correlation Between IL-10 and IL-33 Serum Levels

**Figure 2:**
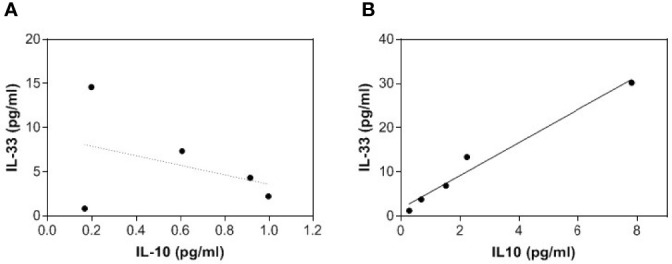
Correlation between IL-33 and IL-10 serum levels. The relationships between IL-33 and IL-10 serum levels both at baseline **(A)** and after 12-month homotaurine treatment **(B)** are reported in the scatterplots, with lines representing linear regression.

After observing an increase in the two anti-inflammatory cytokines IL-33 and IL-10 in treated patents, a correlation between their levels at both T0 and T12 was assessed with the Kendall Rank correlation analysis (**Figure 2**).

While no statistically significant relationships were observed between the levels of the two cytokines at baseline (p=0.476; **Figure 2A**), after 12 months of homotaurine supplementation, a positive correlation was observed between IL-33 and IL-10 levels (Tau=0.44; Tied Z-Value=2.39; p=0.028; **Figure 2B**).

In addition, the delta scores (considered as follow-up values minus baseline values) were calculated for IL-33 and IL-10, and a significant positive correlation was observed also between the delta scores of the two cytokines (Tau=0.80; Tied Z-value=1.960; p=0.05).

### Correlation Between Serum IL-33 and IL-10 Levels and Episodic Memory Performance Scores in MCI Patients

**Figure 3:**
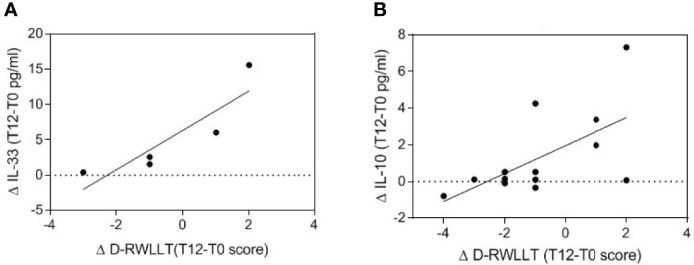
Correlation between serum cytokine changes and episodic memory performances. The relationships between T12-T0 delta values of serum IL-33 (**A**, n=5) or IL-10 (**B**, n=14) and T12-T0 delta scores of delayed recall RWLLT are reported in the scatterplots, with lines representing linear regression.

To measure the episodic memory performance of patients, Rey’s 15-word list learning test was administered as reported in materials and methods section. Delta values, considered as follow-up values minus baseline values, were calculated for IL-33 and IL-10 cytokine levels and for the RWLLT scores and correlated (**Figure 3**).

The Kendall Rank correlation analysis pointed to the existence of a significant relationship between delta values of IL-33 levels and D-RWLLT score (Tau=0.900; Tied Z-value=2.324; p=0.027; **Figure 3A**). Similarly, a significant relationship between the delta values of IL-10 levels and D-RWLLT score was observed (Tau=0.407; Tied Z-value=2.202; p=0.042 **Figure 3B**). In both cases, the increased cytokine levels linked with homotaurine supplementation therapy were correlated with increased episodic memory performance.

No significant correlations were observed between the levels of both cytokines and I-RWLLT scores, including recency and primacy effects (not shown).

Eventually, in substantial accordance with the concept that the modulation of IL-18 system is relevant in homotaurine-mediated effects on episodic memory (16), a significant inverse correlation was shown between delta levels of IL-18BP and I-RWLLT scores (Tau=-0.538; Tied Z-value=-2.712; p=0.007), as well as between delta levels of free IL-18 and I-RWLLT recency effect scores (Tau=-0.549; Tied Z-value=-2.81; p=0.005, **Supplemetal Table 4**).

## Discussion

Neuroinflammation is thought to drive neurodegeneration in a self-reinforcing manner at the earliest period of AD pathogenesis, thus a strong biological rationale inspires the development of therapeutic strategies addressed to target neuroinflammation at preclinical stages (30, 31). A number of neuroimaging and biomarker studies suggest that an inflammatory response occurs both at brain and peripheral level in MCI conditions and increases with disease progression (32, 33), with moderately elevated levels of systemic proinflammatory cytokines that could either mirror the neuroinflammatory status and/or contribute to its development. In any case, exploring potential therapeutic compounds devoid of severe adverse effects and able to enter the brain and modulate the levels of inflammatory cytokines has tremendous implications in AD research and clinical practice.

As described before, homotaurine is a safe and brain permeable compound that can be considered a good candidate for modifying early AD progression. Alongside its reported ability to reduce hippocampal volume loss and ameliorate memory functions in amnestic MCI patients, an anti-inflammatory effect in this clinical context is highly desirable.

Indeed, the present study describes some cytokine modifications in MCI patients treated with homotaurine, which are fully in line with an anti-inflammatory action of the compound.

The main outcome of the study is that a 12-month supplementation of homotaurine in amnestic MCI patients is associated with an increase in the patients’ serum levels of the anti-inflammatory cytokines IL-33 and IL-10, which in turn, appears related to improved episodic memory performances. These findings confirm and expand an our previous study that led us to hypothesize that homotaurine exerts a protective effect in early AD by modulating inflammatory pathways, since MCI patients supplemented with homotaurine showed decreased circulating levels of the pro-inflammatory cytokine IL-18 and improved short-term episodic memory performance as measured by the recency effect of the RWLLT immediate recall. (16),

More in detail, we observed here that in the serum of MCI patients supplemented with homotaurine, beside a decrease in the bioactive form of the pro-inflammatory cytokine IL-18 (**Table 2**), a significant increase in IL-33 and IL-10 (**Table 2** and **Figure 1**) was also evident. Both these cytokines might be considered anti-inflammatory in the studied context. In fact, IL-33 has proposed to hold anti-inflammatory and protective functions in AD (20–22) and IL-10, which can be also induced by IL-33 itself, exerts anti-inflammatory actions and was suggested to negatively regulate amyloid pathology in AD-like conditions (34, 35). The immunomodulating effect of homotaurine was confirmed by the significant association between IL-33 and IL-10 levels observed only after treatment and no at baseline (**Figure 2**). Furthermore, the augmentation of anti-inflammatory cytokines linked to homotaurine treatment is also significantly associated with an improvement in memory functioning, specifically regarding the score of RWLLT delayed recall (**Figure 3**), a neuropsychological tool useful for testing episodic memory and suitable for the cognitive assessment in pre-dementia (36). Notably, the previous and here confirmed finding that the homotaurine-linked decrease of IL-18 in treated MCI is associated with immediate rather than delayed recall tests, opens up an intriguing scenario, in which different stages of verbal episodic memory mapping onto dissociable brain regions (37) might be related to diverse pathways of inflammation. This may have implications for a better understanding of the anatomic basis and the biological correlates of processes underlying episodic memory deficit during early AD.

Albeit we reported in our previous paper that homotaurine treatment was effective in lowering circulating IL-18 levels only in MCI patients carrying the APOEϵ4 allele, in the present study we did not select the patients based on their genotype because of their small number. However, we could not exclude a potential effect of APOE gene variants on IL-33 and IL-10 results.

Eventually, as for the potential molecular mechanisms underlying the homotaurine anti-inflammatory effects exerted in MCI patients, it is important to highlight that homotaurine is a GABA-receptor agonist and GABA-mediated mechanisms are both crucial for learning and memory tasks and able to suppress the inflammatory response of astrocytes and microglia (11). In addition, through GABA-mediated pathways homotaurine has anti-inflammatory and therapeutic properties in a multiple sclerosis animal model, even involving increased IL-10-secreting responses (13, 14), but unfortunately IL-18 and IL-33 cytokines have not been evaluated in that context. Overall, more specific studies should be designed to address the homotaurine mechanism of action as regards in particular its impact on inflammation in early AD.

Our study should be considered preliminary, since it was performed with a small number of patients and the generalizability of the results we obtained is limited, but our findings are intriguing. They support the notion that homotaurine supplementation is suitable for treatment of MCI patients and strongly suggest that its use can modulate serum cytokine levels and regulate the pro/anti-inflammation balance, in association with the improvement of episodic memory. These results encourage performing larger longitudinal and placebo-controlled studies to confirm the efficacy of homotaurine supplementation for treatment of MCI patients and to explore its potential to target inflammation. If the data we obtained here will be confirmed, then they would offer major advance in the battle against AD.

## Data Availability Statement

The raw data supporting the conclusions of this article will be made available by the authors, without undue reservation.

## Ethics Statement

The studies involving human participants were reviewed and approved by Ethic Committee of Fondazione Santa Lucia. The patients/participants provided their written informed consent to participate in this study.

## Author Contributions

PB and GS designed the study. NB and GS performed clinical assessment. ET, BM, LS, and SG collected samples and performed experiments. ET, GS, and PB analyzed and interpreted the data. ET, LS, GS, and PB made a substantial, direct and intellectual contribution to the article designing and wrote the draft. NB, BM, LS, SG, and ML contributed to writing and revising the article. All authors have reviewed and approved the final version of the manuscript for publication.

## Funding

This project was supported by the Italian Ministry of Health (RC 2018-2021; ADIMB project, CoEN 2019-Pathfinder IV).

## Conflict of Interest

The author GS consulted for Neuraxpharm in 2019, on the role of nutraceuticals in neurodegenerative dementias.

The remaining authors declare that the research was conducted in the absence of any commercial or financial relationships that could be construed as a potential conflict of interest.

The reviewer VC declared a shared affiliation with the authors to the handling editor at the time of review.

## Publisher’s Note

All claims expressed in this article are solely those of the authors and do not necessarily represent those of their affiliated organizations, or those of the publisher, the editors and the reviewers. Any product that may be evaluated in this article, or claim that may be made by its manufacturer, is not guaranteed or endorsed by the publisher.
