# Supplementary material for: IL-33 and IL-10 Serum Levels Increase in MCI Patients Following Homotaurine Treatment
Source: Front Immunol. 2022 Apr 19;13:813951. doi: 10.3389/fimmu.2022.813951 (PMC9061963; doi:10.3389/fimmu.2022.813951)
Supplement: Supplementary file 1 [file Table_1.pdf]

Table S1. Sociodemographic and clinical characteristics of included MCI patients

					***Baseline** (T0)*						***After 12 month treatment** (T12)*					
Individuals	***Gender***	***Age***	***Education***	***ApoE***	***IADL***	***MMSE***	***I-RWLLT***	***D-RWLLT***	***Primacy***	***Recency***	***IADL***	***MMSE***	***I-RWLLT***	***D-RWLLT***	***Primacy***	***Recency***
1	f	79	10	E3/E4	11	26	16	3	8	2	10	25	18	1	7	7
2	f	72	5	E3/E4	8	28	25	4	9	11	15	28	30	6	9	12
3	m	70	8	E3/E4	7	24	38	6	14	13	6	26	28	2	14	6
4	m	72	8	E3/E3	9	28	29	3	13	12	11	24	29	4	9	16
5	f	76	6	E3/E3	8	23	27	4	17	10	12	23	19	1	5	14
6	m	78	13	E3/E3	5	27	24	5	9	13	5	27	20	3	1	12
7	f	85	13	E3/E4	15	26	25	1	9	15	15	26	14	0	2	17
8	m	78	13	E3/E3	5	28	20	1	5	13	7	26	24	0	9	14
9	f	72	6	E3/E3	14	29	33	6	16	11	12	27	36	8	12	14
10	f	79	5	E3/E4	17	27	18	2	12	6	12	25	20	0	12	6
11	f	86	12	E3/E3	9	29	32	5	13	11	10	29	20	4	6	10
12	f	78	6	E3/E3	10	28	31	4	11	13	11	27	26	3	13	9
13	m	71	17	E3/E4	5	30	24	4	7	12	6	27	28	2	4	18
14	m	66	13	E4/E4	5	26	35	1	17	10	7	26	28	2	6	18

MMSE, Mini-Mental State Examination; IADL, Instrumental Activities of Daily Living; I-RWLLT, Immediate recall Rey 15-Word List Learning Test; D-RWLLT, Delayed recall Rey 15-Word List Learning Test.

Table S2. Cytokine levels in included MCI patients

	**Baseline** (T0)							**After 12 month treatment** (T12)						
**Individuals**	**IL-1 β**	**Tot IL-18**	**IL-18 BP**	**Free IL-18**	**IL-1 RA**	**IL-33**	**IL-10**	**IL-1 β**	**Tot IL-18**	**IL-18 BP**	**Free IL-18**	**IL-1 RA**	**IL-33**	**IL-10**
1	0.000	106	12339	63	361	0.000	0.607	0.000	100	13072	58	333	0.000	0.510
2	0.387	264	10975	164	327	0.000	0.325	1.453	260	10835	163	307	0.000	0.396
3	0.000	56	17008	29	170	0.000	0.914	0.000	104	19610	49	189	0.000	0.128
4	1.454	151	8772	101	223	0.000	0.125	0.000	137	13827	77	218	0.000	3.503
5	0.779	192	12549	113	269	0.860	0.167	1.055	152	15026	83	269	1.279	0.278
6	0.006	463	11701	283	189	0.000	0.062	0.000	469	13943	266	149	0.000	0.108
7	0.364	165	8668	111	383	2.240	1.018	0.075	151	10870	94	199	3.821	0.683
8	1.125	335	12717	197	279	4.339	0.996	0.824	309	13355	178	125	6.927	1.516
9	1.913	208	13760	118	408	14.610	0.501	0.392	205	15292	111	236	30.212	7.810
10	0.050	153	17273	78	385	0.000	0.108	0.000	141	18839	69	382	0.000	0.250
11	0.234	101	18421	50	293	0.000	0.197	0.122	125	21154	57	350	0.000	0.305
12	0.000	443	17648	225	600	0.000	0.268	0.000	474	19627	228	737	0.000	4.520
13	0.000	486	28741	188	426	0.000	0.192	0.922	220	21810	99	725	0.000	0.710
14	0.614	342	26042	140	1837	7.364	0.250	2.511	263	22921	116	558	13.420	2.227

IL-18 BP, Interleukin-18 Binding Protein; Free IL-18, IL-18 BP-unbound IL-18; IL-1RA, IL-1 Receptor Antagonist. All cytokines are measured as pg/ml

TABLE S3. Correlations between IADL scores and cytokine levels

	Baseline (T0)			After 12 month treatment (T12)		
	p value	***Tau***	***Tied Z Value***	***p value***	***Tau***	***Tied Z Value***
**IADL scores and Cytokine levels**						
IL-1β	0.9563	0.011	0.059	0.6222	0.099	0.565
Total IL-18	0.0897	-0.341	-1.777	0.8695	-0.033	-0.172
IL-18 BP	0.2983	-0.209	-1.089	0.0897	-0.341	-1.777
Free IL-18	0.1124	-0.319	-1.662	0.9563	0.011	0.057
IL-1RA	0.2983	0.209	1.089	0.8695	0.033	0.172
IL-33*	0.4434	0.154	0.985	0.8267	0.044	0.293
IL-10	0.6222	0.099	0.516	0.3520	0.187	0.974

	p value	***Tau***	***Tied Z Value***
**Δ IADL scores (T12-T0) and Δ Cytokines levels (T12-T0)**			
IL-1β	0.4641	0.154	0.822
Total IL-18	>0.999	0.00	0.00
IL-18 BP	0.7016	0.077	0.401
Free IL-18	0.8695	0.033	0.174
IL-1RA	0.8695	0.033	0.172
IL-33*	0.6242	-0.200	-0.548
IL-10	0.7016	0.077	0.401

*Measurable only in serum of 5 patients out of 14

TABLE S4. Correlations between changes in episodic memory performances and cytokine levels

	p value	*Tau*	*Tied Z Value*
**Δ I-RWLLT scores (T12-T0) and Δ Cytokine levels (T12-T0)**			
IL-1β	0.9128	0.022	0.113
Total IL-18	0.3244	-0.198	-1.002
IL-18 BP	0.0070	-0.538	-2.712
Free IL-18	0.5470	-0.121	-0.616
IL-1RA	0.7016	-0.077	-0.387
IL-33*	0.3272	0.400	0.980
IL-10	0.2503	0.231	1.162
**Δ D-RWLLT scores (T12-T0) and Δ Cytokine levels (T12-T0)**			
IL-1β	0.2284	-0.242	-1.331
Total IL-18	0.9128	-0.022	-0.120
IL-18 BP	0.3520	-0.187	-1.012
Free IL-18	>0.999	0.000	0.000
IL-1RA	0.0897	-0.341	-1.845
IL-33*	0.0275	0.900	2.324
IL-10	0.0428	0.407	2.202
**Δ Primacy scores (T12-T0) and Δ Cytokine levels (T12-T0)**			
IL-1β	0.6222	-0.099	-0.515
Total IL-18	0.3520	0.187	0.963
IL-18 BP	0.3811	-0.176	-0.901
Free IL-18	0.1005	0.330	1.708
IL-1RA	0.2736	0.220	1.126
IL-33*	0.3272	0.400	0.980
IL-10	0.8267	0.044	0.225
**Δ Recency scores (T12-T0) and Δ Cytokine levels (T12-T0)**			
IL-1β	0.622	0.099	0.510
Total IL-18	0.0008	-0.670	-3.415
IL-18 BP	0.0798	-0.352	-1.781
Free IL-18	0.0062	-0.549	-2.815
IL-1RA	0.1889	-0.264	-1.336
IL-33*	>0.999	0.000	0.000
IL-10	0.3244	0.198	1.002

*Measurable only in serum of 5 patients out of 14
